# A sequence polymorphism on 8q24 is associated with survival in hepatocellular carcinoma patients who received radiation therapy

**DOI:** 10.1038/s41598-018-20700-x

**Published:** 2018-02-02

**Authors:** Xiao-Mei Zhao, Zuo-Lin Xiang, Yi-Xing Chen, Ping Yang, Yong Hu, Zhao-Chong Zeng

**Affiliations:** Departments of Radiation Oncology, Zhongshan Hospital, Fudan University, Shanghai, China

## Abstract

There is a growing consensus that genetic variation in candidate genes can influence cancer progression and treatment effects. In this study, we genotyped the rs9642880 G > T polymorphism using DNA isolated from blood samples of 271 hepatocellular carcinoma (HCC) patients who received radiotherapy treatment. We found that patients who carried the GT or TT genotypes had significantly shorter median survival times (MSTs) compared to patients with the GG genotype (14.6 vs.21.4 months). The multivariate *P* value was 0.027, the hazard ratio (HR) was 1.38, and the 95% confidence interval was 1.04–1.84. Further analysis revealed that patients with the variant genotypes had an increased risk of poor tumour response to radiotherapy (*P* = 0.036 and 0.002 for stable disease and progressive disease, respectively) and higher incidence of multiple intrahepatic lesions (*P* = 0.026) and BCLC C stage (*P* = 0.027). Moreover, further stratified survival analyses revealed that at least radioresponse and BCLC stage contributed to the association between the rs9642880 G > T polymorphism and survival of HCC patients in this study (P value, 0.017 *vs* 0.053 for BCLC C stage *vs* B stage; 0.011 *vs* 0.531 for radioresponse SD + PD *vs* CR + PR). These results illustrate the potential association between rs9642880 G > T and survival in HCC patients who received radiotherapy treatment.

## Introduction

Hepatocellular carcinoma (HCC) is one of the most common liver malignancies, and its incidence is increasing in most parts of the world. Surgical resection is the most common treatment choice for HCC, but for a considerable number of cases, surgical resection is not suitable because of factors such as large tumour size, concomitant liver cirrhosis, or portal vein tumour thrombosis. In these inoperable HCC cases, radiotherapy can be an effective treatment option^1,2^.

In clinical radiobiology, HCC is an early-responding tissue with an α/β ratio >10 Gy, which means it is categorized as a radiation-sensitive tissue. However, historically, HCC radiotherapy has been limited by the lack of precise patient imaging, the inability to quantify the dose received by a given volume of tissue, and outmoded radiation therapy (RT) techniques. More recently, the development of modern RT techniques, such as helical tomotherapy (HT), has made radiation therapy for HCC a safe and effective option for many patients.

Genetic variations, such as single nucleotide polymorphisms (SNPs) in candidate genes, have been proposed to affect the survival outcomes in cancer patients^3,4^. In this study, we investigated the rs9642880 G > T polymorphism, which is located on 8q24, only 30 kb upstream of the *c-MYC*(*MYC*) gene. c-MYC is a transcription factor that regulates many basic cellular processes, such as cell proliferation, cell transformation, and apoptosis^5,6^, and the deregulation of c-MYC plays a critical role in carcinogenesis and tumour progression.

Additionally, according to previous studies, c-MYC has been revealed to be a radiosensitive locus^7^, and it has been found that the rs9642880 G > T polymorphism could influence the expression of *c-MYC*^8^. The overexpression of c-MYC is considered to be associated with promoting radioresistance in nasopharyngeal carcinomas^9^; therefore, we hypothesize that the *c-MYC* rs9642880 G > T polymorphism is associated with the survival of HCC patients who received radiation therapy. To confirm this hypothesis, we evaluated the frequencies of the rs9642880 G > T polymorphism in HCC patients who received radiotherapy treatment and assessed survival outcome and the impact of genotype for this polymorphism on clinical characteristics.

## Results

### Patient demographic and clinical information and outcomes

The demographic information and clinical characteristics of the 271 HCC patients included in this study are summarized in Table 1. The study cohort was composed of 227 men and 44 women, and the median age was 56 years (ages ranged from 26 to 87 years). Additional demographic information and clinical characteristics included Karnofsky performance status(KPS), alpha-fetoprotein(AFP), Child-Pugh classification, Barcelona Clinic Liver Cancer stage(BCLC stage), tumour size, intrahepatic tumour number, prior treatments, radiation technique, lymph node metastases(LN metastases), distant metastases, smoking status and drinking status (Table 1). At the end of the study, 53 patients (19.6%) were still alive. However, among those patients, only 35 were available for follow-up. Eight patients were lost to death unrelated to HCC, and ten patients were lost due to loss of contact. Of the 218 patients (80.4%) who died over the course of the study, 159 (72.9%) died from hepatic failure due to hepatic decompensation or tumour progression. Severe lung, brain, or bone metastases were observed in 59 patients (27.1%). Other causes of death included gastrointestinal bleeding in four patients and systemic failure in two patients.

**Table 1:** Demographic and clinical characteristics of the 271 HCC patients.

Variables	n	%
Age (y)		
Average	55.88 ± 0.708	
median	56	
Gender		
Male	227	83.76
Female	44	16.24
KPS		
100	28	10.33
90	223	82.29
80	20	7.38
AFP		
<20	98	36.16
≥20	173	63.84
Child-Pugh classification		
A	228	84.13
B	43	15.87
BCLC stage		
B	149	54.98
C	122	45.02
Tumour Size		
<5	94	34.69
≥5	177	65.31
Intrahepatic tumour number		
Solitary	128	47.23
Multiple	143	52.77
Prior treatments		
None	29	10.70
Operation ± TACE	101	37.27
TACE only	141	52.03
Radiation technique		
3D-CRT	135	49.82
HT	136	50.18
LN metastases		
Absence	207	76.38
Presence	64	23.62
Distant metastases		
Absence	253	93.36
Presence	18	6.64
Smoking status		
Smokers	156	57.6
Non-smokers	115	42.4
Drinking status		
Drinker	144	53.1
Non-drinker	127	46.9

### Associations between patient characteristics and HCC survival outcomes

Analyses of potential predictors of survival in the 271 HCC patients are summarized in Table 2. A Cox proportional hazards regression model was used to identify predictors of survival. In the univariate analysis, we found that alanine aminotransferase (ALT, *P* = 0.017), aspartate aminotransferase (AST, *P* = 0.018), AFP (*P* = 0.031), Child-Pugh classification (*P* = 0.001), BCLC stage (*P* = 0.003), intrahepatic tumour number (*P* < 0.001), external beam radiotherapy (EBRT) dose (*P* = 0.029), and radiation technique (*P* = 0.025) were significantly associated with overall survival(OS) (Table 2). Based on these findings, factors that significantly predicted survival in the univariate analysis were used to adjust the multivariate Cox regression to eliminate possible interference with the main effects of the rs9642880 G > T polymorphism on HCC prognosis (Table 3).

**Table 2:** Univariate analyses of potential predictors of survival in 271 HCC patients.

Variables	n	Survival status	Overall survival analyses		
			Log-rank *P*	HR(95%CI)	*P*
Age (y)			0.945		
<60	177	16.3 ± 1.12		1	
≥60	94	16.9 ± 1.98		0.99(0.75–1.31)	0.945
Gender			0.853		
Female	44	16.9 ± 2.33		1	
Male	227	16.1 ± 1.12		1.03(0.72–1.48)	0.853
HBsAg			0.059		
Negative	30	20.8 ± 2.89		1	
Positive	241	16.0 ± 1.06		1.53(0.98–2.39)	0.061
ALT (IU/ml)			**0**.**016**		
<75	231	16.7 ± 1.08		1	
≥75	40	14.6 ± 2.58		1.54(1.08–2.20)	**0**.**017**
AST (IU/mL)			**0**.**017**		
<75	233	16.9 ± 1.05		1	
≥75	38	13.2 ± 1.89		1.56(1.08–2.26)	**0**.**018**
AFP			**0**.**030**		
<20	98	19.7 ± 2.00		1	
≥20	173	15.2 ± 1.10		1.37(1.03–1.81)	**0**.**031**
Child-Pugh classification			**0**.**001**		
A	228	17.4 ± 1.28		1	
B	43	14.4 ± 2.47		1.80(1.26–2.56)	**0**.**001**
BCLC stage			**0.002**		
B	149	20.8 ± 1.19		1	
C	122	13.1 ± 1.28		1.51(1.15–1.98)	**0**.**003**
Tumour Size			0.514		
<5	94	16.9 ± 1.55		1	
≥5	177	16.1 ± 1.54		0.91(0.69–1.21)	0.515
Intrahepatic tumour number			**<0**.**001**		
Solitary	128	23.4 ± 1.96		1	
Multiple	143	12.4 ± 1.52		2.32(1.76–3.05)	**<0**.**001**
Prior treatments			0.210		
None	29	15.2 ± 4.55		1	
TACE only	141	16.1 ± 0.92		0.85(0.55–1.32)	0.469
Operation ± TACE	101	17.8 ± 2.15		0.72(0.46–1.14)	0.158
EBRT dose (Gy)			**0**.**028**		
≤50	137	15.0 ± 0.80		1	
>50	134	18.7 ± 1.91		0.74(0.57–0.97)	**0**.**029**
Radiation technique			**0**.**025**		
3D-CRT	135	15.2 ± 1.28		1	
HT	136	18.4 ± ‘1.75		1.36(1.04–1.78)	**0**.**025**
LN metastases			0.106		
Absence	207	17.0 ± 1.23		1	
Presence	64	15.2 ± 1.30		1.29(0.95–1.77)	0.108
Distance metastases			0.276		
Absence	253	16.9 ± 0.98		1	
Presence	18	11.0 ± 1.78		1.37(0.78–2.40)	0.279

Bold values indicate statistical significance (P < 0.05).

**Table 3:** Association between rs9642880 G > T genotypes and overall survival of HCC patients.

genotypes	n	Survival status(mo)^a^	Log-rank *P*	Univariate	*P*	Multivariate	*P* ^b^
				HR(95% CI)		HR(95% CI)^b^	
general			**0**.**003**				
GG	105	21.4 ± 1.88		1		1	
GT	130	14.7 ± 0.64		1.53(1.14–2.05)	**0**.**004**	1.33(0.99–1.80)	0.059
TT	36	13.0 ± 3.21		1.96(1.26–3.02)	**0**.**003**	1.69(1.07–2.68)	**0**.**026**
Dominant			**0**.**001**				
GG	105	21.4 ± 1.88		1		1	
GT + TT	166	14.6 ± 0.76		1.58(1.20–2.09)	**0**.**001**	1.38(1.04–1.84)	**0**.**027**

Bold values indicate statistical significance (P < 0.05).

^a^Survival status is expressed as the median survival time ± standard error. Abbreviations: mo = months.

^b^Adjusted by ALT, AST, AFP, Child-Pugh classification, BCLC stage, Intrahepatic tumour number, EBRT dose and radiation technique.

### Associations between rs9642880 G > T and HCC survival outcomes

**Figure 1:**
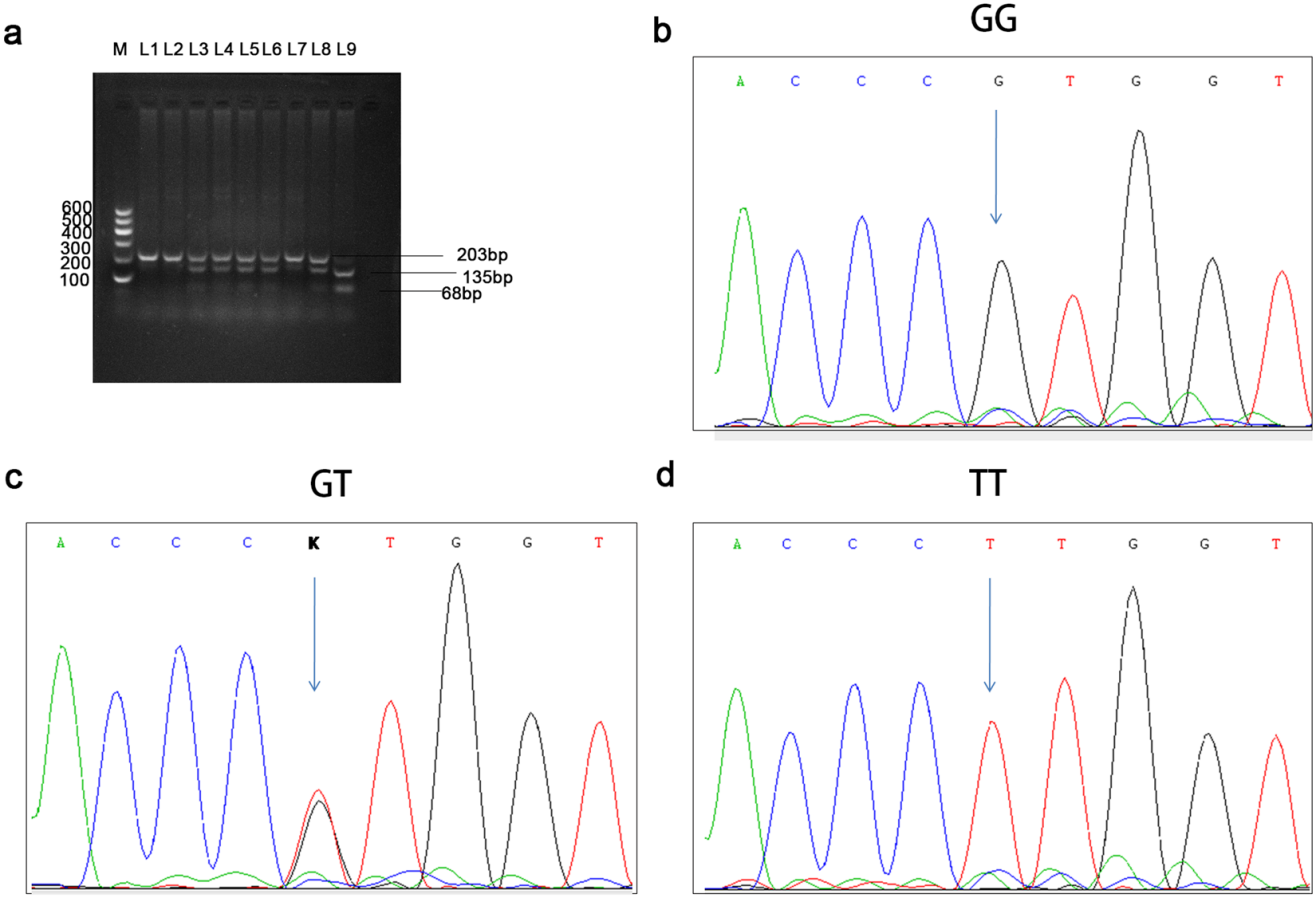
rs9642880 G > T genotypes were identified using a polymerase chain reaction-restriction fragment length polymorphism (PCR-RFLP) assay, and the results were confirmed using direct sequencing. (**a**) Representative genotypes based on the PCR-RFLP assay. L1, L2, and L7 were identified as having the GG genotype; L3, L4, L5, L6, and L8 were identified as having the GT genotype; and L9 was identified as having the TT genotype. The figure of the full-length agarose gel and figure with white background and black product bands is included in the Supplementary Figs 2a and 3a; (**b**,**c** and **d**) Results of direct DNA sequencing of L1, L3, and L9, respectively.

**Figure 2:**
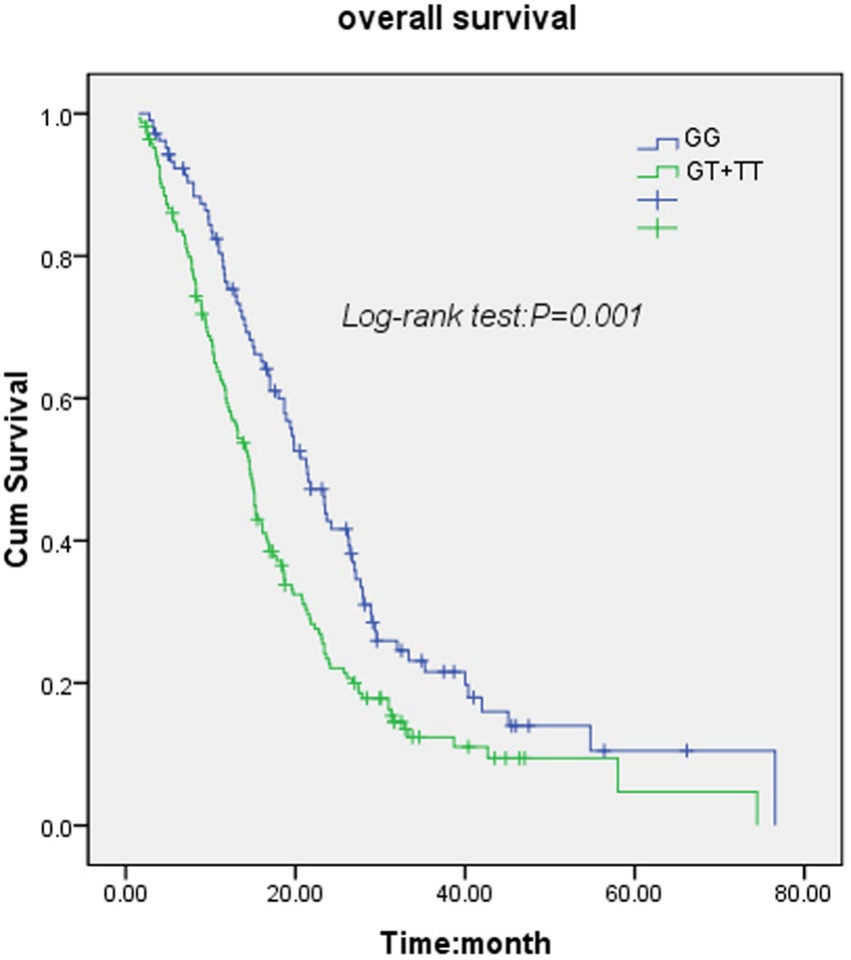
Kaplan-Meier survival curves of 271 HCC patients based on rs9642880 G > T genotype (GT + TT *vs* GG).

The rs9642880 G > T genotypes were identified using the polymerase chain reaction-restriction fragment length polymorphism (PCR-RFLP) assay and verified by direct sequencing (Fig. 1). There were 105 HCC patients with the GG genotype, 130 patients with the GT genotype, and 36 patients with the TT genotype. As shown in Table 3, rs9642880 G > T was significantly associated with OS in HCC patients with radiotherapy treatment (log-rank *P* = 0.003). Patients who carried the TT variant genotype had significantly shorter median survival times (MSTs) compared with those with the GG genotype (13.0 vs.21.4 months), with a univariate *P* value of 0.003 (HR = 1.96; 95% confidence interval [CI] 1.26–3.02) and a multivariate *P* value of 0.026 (HR = 1.69; 95% CI 1.07–2.68). The MST of patients who carried the GT or TT genotype was shorter than that of patients with the GG genotype (14.6 vs. 21.4 months), with a univariate *P* value of 0.001 (HR = 1.58, 95% CI 1.20–2.09) and a multivariate *P* value of 0.027 (HR = 1.38; 95% CI 1.04–1.84) (Table 3). These results demonstrate that patients with at least one T allele (GT or TT genotypes) had significantly poorer survival compared with those carrying the GG homozygous genotype (log-rank *P* = 0.001, Fig. 2).

### Association between the rs9642880 G > T polymorphism and the clinical characteristics of HCC patients

We also analysed the association between the rs9642880 G > T polymorphism and the clinical characteristics of HCC patients. In contrast to patients with the GG genotype, patients with GT or TT genotypes had a higher incidence of multiple intrahepatic lesions (*P* = 0.026) and BCLC C stage (*P* = 0.027) and an increased risk of stable disease (SD) or progressive disease (PD) after tumour radiotherapy (*P* = 0.036 and *P* = 0.002, respectively). No significant correlation was observed with other clinical characteristics (Table 4).

**Table 4:** Associations between rs9642880 G > T and demographic and clinical characteristics.

variable	GT + TT	GG	Univariate	*P*	Multivariate	*P* ^a^
			RR(95% CI)		RR(95%CI)^a^	
Age(y)						
<60	105(63.3%)	72(68.6%)	1		1	
≥ 60	61(36.7%)	33(31.4%)	1.27(0.75–2.13)	0.371	1.38(0.81–2.37)	0.242
Gender						
Female	25(15.1%)	19(18.1%)	1		1	
Male	141(84.9%)	86(81.9%)	1.25(0.65–2.40)	0.510	1.10(0.56–2.15)	0.792
HBsAg						
Negative	18(10.8%)	12(11.4%)	1		1	
Positive	148(89.2%)	93(88.6%)	1.06(0.49–2.30)	0.881	1.00(0.45–2.25)	0.987
ALT(IU/ml)						
<75	139(83.7%)	92(87.6%)	1		1	
≥75	27(16.3%)	13(12.4%)	1.38(0.67–2.80)	0.381	0.70(0.30–1.60)	0.396
AST (IU/ml)						
<75	138 (83.1%)	95(90.5%)	1		1	
≥75	28(16.9%)	10(9.5%)	1.93(0.89–4.15)	0.094	2.31(0.95–5.61)	0.066
AFP						
<20	58(34.9%)	40(38.1%)	1		1	
≥20	108(65.1%)	65(61.9%)	1.15(0.69–1.90)	0.598	1.00(0.59–1.69)	0.997
Child-Pugh classification						
A	138(83.1%)	90(85.7%)	1		1	
B	28(16.9%)	15(14.3%)	1.22(0.62–2.41)	0.571	1.14(0.57–2.29)	0.714
BCLC stage						
B	82(49.4%)	68(64.8%)	1		1	
C	84(50.6%)	37(35.2%)	1.88(1.14–3.11)	**0.014**	1.83(1.07–3.12)	**0**.**027**
Tumour Size						
<5	61(36.7%)	33(31.4%)	1		1	
≥5	105(63.3%)	72(68.6%)	0.79(0.47–1.33)	0.371	0.88(0.52–1.50)	0.638
Intrahepatic tumour number						
Solitary	69(41.6%)	59(56.2%)	1		1	
Multiple	97(58.4%)	46(43.8%)	1.80(1.10–2.96)	**0**.**019**	1.80(1.07–3.01)	**0**.**026**
Prior treatments						
None	18(10.8%)	11(10.5%)	1		1	
TACE only	87(52.4%)	54(51.4%)	0.99(0.43–2.24)	0.970	0.79(0.33–1.90)	0.600
Operation ± TACE	61(36.7%)	40(38.1%)	0.93(0.40–2.18)	0.871	1.00(0.40–2.52)	0.994
EBRT dose (Gy)						
≤50	87(52.4%)	50(47.6%)	1		1	
>50	79(47.6%)	55(52.4%)	0.83(0.50–1.35)	0.442	0.92(0.56–1.52)	0.749
Radiation technique						
HT	81(48.8%)	55(52.4%)	1		1	
3D-CRT	85(51.2%)	50(47.6%)	1.15(0.71–1.88)	0.565	1.21(0.73–2.01)	0.463
LN metastases						
Absence	128(77.1%)	79(75.2%)	1		1	
Presence	38(22.9%)	26(24.8%)	0.90(0.51–1.60)	0.724	0.68(0.38–1.23)	0.204
Distant metastases						
Absence	158(95.2%)	95(90.5%)	1		1	
Presence	8(4.8%)	10(9.5%)	0.48(0.18–1.26)	0.137	0.43(0.17–1.09)	0.076
Response to EBRT						
CR + PR	60(36.1%)	65(61.9%)	1		1	
SD	46(27.7%)	22(21.0%)	2.27(1.22–4.20)	**0**.**009**	1.98(1.05–3.74)	**0**.**036**
PD	60(36.1%)	18(17.1%)	3.61(1.92–6.80)	**<0**.**001**	3.01(1.51–6.02)	**0**.**002**

Bold values indicate statistical significance (P < 0.05).

^a^Adjusted by ALT, AST, AFP, Child-Pugh classification, BCLC stage, Intrahepatic tumour number, EBRT dose, Radiation technique.

### Stratified survival analyses for rs9642880 G > T

**Figure 3:**
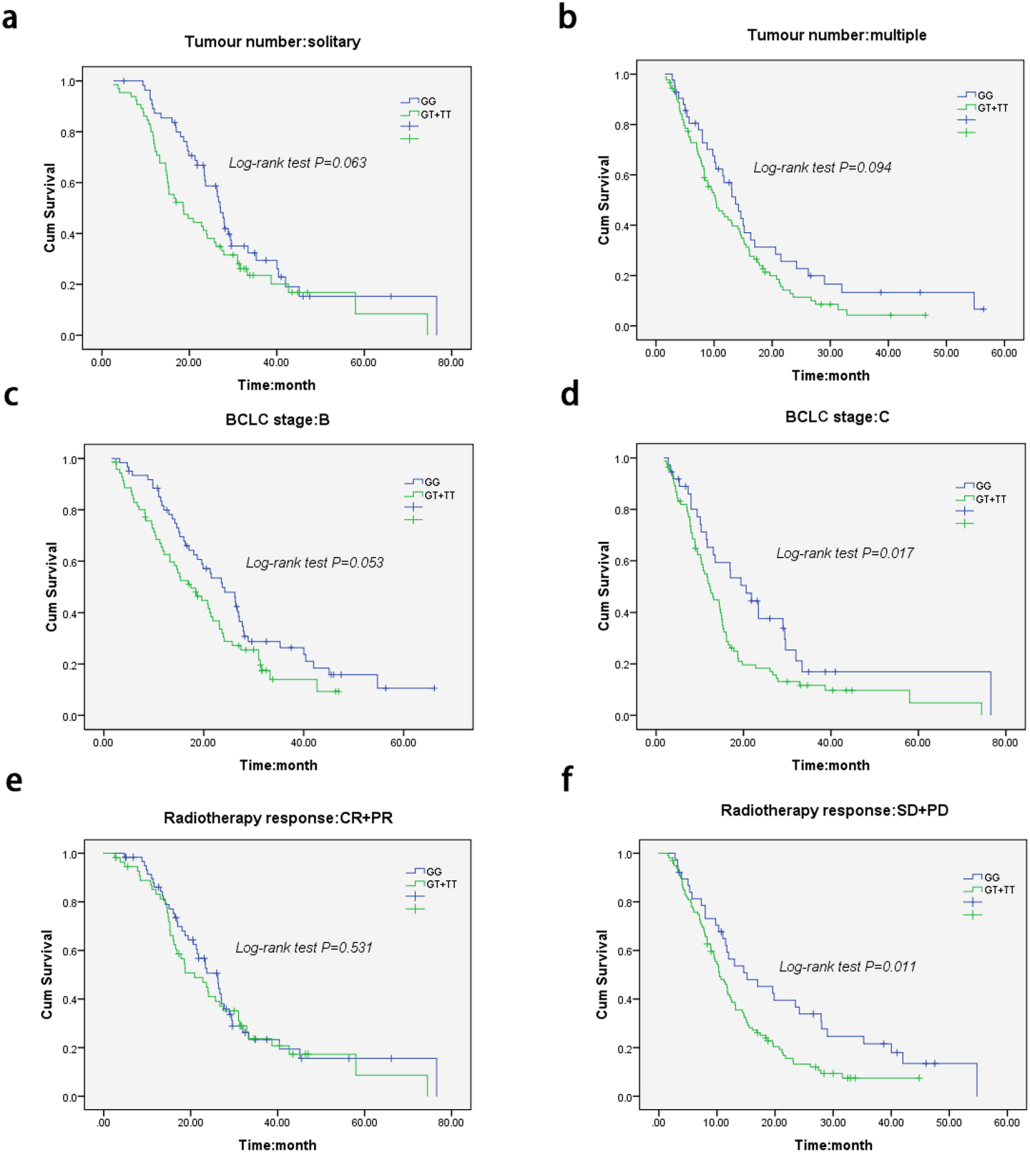
Stratified Kaplan-Meier survival curves of HCC patients who received radiotherapy, based on the rs9642880 G > T genotype. (**a**) Kaplan-Meier survival curves of HCC patients with solitary intrahepatic tumour; (**b)** Kaplan-Meier survival curves of HCC patients with multiple intrahepatic tumours; (**c)** Kaplan-Meier survival curves of HCC patients in BCLC B stage; (**d)** Kaplan-Meier survival curves of HCC patients in BCLC C stage; (**e)** Kaplan-Meier survival curves of HCC patients with CR or PR radioresponse; (**f)** Kaplan-Meier survival curves of HCC patients with SD or PD radioresponse.

To determine the clinical characteristics that contribute to the association between the rs9642880 G > T polymorphism and survival in HCC patients who received radiation therapy in the study, stratified survival analyses by intrahepatic tumour number (solitary *vs* multiple), BCLC stage (B stage *vs* C stage), and radioresponse (CR + PR *vs* SD + PD) with the rs9642880 genotypes were carried out. The log-rank *P* value was 0.063 in the solitary subgroup (Fig. 3a), 0.094 in the multiple subgroup (Fig. 3b), 0.053 in the BCLC B stage subgroup (Fig. 3c), 0.017 in the BCLC C stage subgroup (Fig. 3d), 0.531 in the CR + PR radioresponse subgroup (Fig. 3e), and 0.011 in the SD + PD radioresponse subgroup (Fig. 3f). A significantly poor survival status associated with the variant genotypes was found in BLBC C stage patients and poor radioresponse patients but not in BLBC B stage and good radioresponse patients (P value, 0.017 *vs* 0.053 for BCLC C stage *vs* B stage; 0.011 *vs* 0.531 for radioresponse SD + PD *vs* CR + PR). However, no significant differences related to intrahepatic tumour number were found; these results indicate that at least radioresponse and BCLC stage contribute to the association between the rs9642880 G > T polymorphism and survival of HCC patients who received radiation therapy in the study.

### Association between the rs9642880 G > T polymorphism and c-MYC expression in HCC cell lines

We analysed the genotype of the rs9642880 G > T polymorphism in different HCC cell lines, including MHCC97H, HCCLM3,HepG2, Hep3B and SMMC-7721, and found that cell lines MHCC97H and HCCLM3 had the GG genotype, HepG2 had the GT genotype, and Hep3B and SMMC-7721 had the TT genotype (Supplementary Fig. 1a). Additionally, we quantified the c-MYC mRNA levels in those HCC cell lines using a quantitative real-time PCR assay, and the results showed that the cell lines with the TT genotype (Hep3B and SMMC-7721) had higher c-MYC mRNA levels compared with the cell line with the GT genotype (HepG2); moreover, the cell lines with the GG genotype (MHCC97H and HCCLM3) had the lowest c-MYC mRNA level (Supplementary Fig. 1b) of the five cell lines. These results were confirmed at the protein level by western blot assays (Supplementary Fig. 1c) and suggested that the rs9642880 T allele was associated with the overexpression of c-MYC in HCC cell lines.

## Discussion

Currently, genome-wide association studies are widely undertaken to develop more accurate diagnostic and therapeutic strategies for human diseases, including cancer^10,11^. In the present study, we investigated the associations between the *c-MYC* rs9642880 G > T polymorphism on 8q24 and survival in 271 HCC patients following radiotherapy treatment. We found that the presence of at least one T allele was significantly associated with an unfavourable survival outcome for HCC patients following radiation therapy. These results remained significant after adjusting for other potential survival-influencing factors. Moreover, further analyses revealed that three clinical characteristics were associated with the rs9642880 G > T polymorphism: tumour response to radiotherapy, intrahepatic tumour number and BCLC stage; patients with the GT or TT genotype had an increased risk of poor tumour response to radiotherapy and a higher incidence of multiple intrahepatic lesions and BCLC C stage. However, the further stratified analyses emphasized the contribution of radioresponse and BCLC stage, but not intrahepatic tumour number, to the association between the rs9642880 G > T polymorphism and survival of HCC patients. These findings indicate the potential association between rs9642880 G > T and the survival of HCC patients who received radiotherapy treatment.

The incidence of HCC is increasing in most parts of the world, and clinical data indicate that radiotherapy is a highly effective therapy^1,12–14^. However, we found that HCC patients at the same disease stage often have completely different outcomes following radiotherapy, which is believed to be due to individual variation in radiation sensitivity. There is an increasing consensus that radiation sensitivity is a genetic trait that involves the interactions of many genes^15^; thus, genetic variants may influence an individual’s radioresponse.

The underlying mechanism of radiotherapy is the production of irreparable DNA damage, which leads to cell death. Cells respond to DNA damage by activating DNA repair pathways, including cell cycle checkpoints and other cell cycle regulatory pathways. Therefore, the status of DNA damage or DNA repair pathways is one of the most important determinants of radiosensitivity^16,17^. Interestingly, c-MYC is involved in cell cycle checkpoint regulation. Wang and colleagues found that the overexpression of c-MYC activated DNA damage cell cycle checkpoints by upregulating the expression of the CHK1 and CHK2 checkpoint kinases in nasopharyngeal carcinomas, leading to the activation of DNA repair and ultimately resulting in radioresistance^9^.

The rs9642880 G > T polymorphism is a common variant that lies upstream of *c-MYC* on chromosome 8q24^18,19^.Various enhancers that can regulate the transcription of c-*MYC* are found in this region, and the rs9642880 G > T polymorphism has been reported to affect the expression of c-MYC in bladder cancer. It was found that c-MYC expression levels were significantly higher in individuals with the rs9642880 GT or TT genotype than in those with the GG genotype^8^, which is consistent with our analysis of c-MYC expression in HCC cell lines with different rs9642880 genotypes. Thus, the rs9642880 G > T polymorphism may influence radiosensitivity by altering c-MYC expression, ultimately leading to radioresistance. Moreover, *c-MYC* has been revealed to be a radiosensitive locus^13^, and genetic alteration in that region may influence radiosensitivity. In the present study, we confirmed that HCC patients with the rs9642880 GT or TT genotypes have poorer radioresponse. Further stratified analyses revealed that radioresponse contributed to the influence of the rs9642880 G > T polymorphism on survival.

c-MYC is a transcription factor that regulates many basic cellular processes, such as cell proliferation, cell transformation, and apoptosis^5,6^. Given its role in these basic cellular functions, the deregulation of c-MYC plays a critical role in carcinogenesis and tumour progression. For example, Kim *et al*. found that the c-MYC regulatory network accounts for similarities between embryonic stem and cancer cell transcription programs^20^. Additionally, Castro *et al*. reported that c-MYC activates GATA4, which leads to metastasis in lung adenocarcinoma^21^. In HCC, c-MYC is intimately involved in malignant progression; in particular, c-MYC has been implicated in driving the initial stages of hepatocarcinogenesis^22^ and is commonly overexpressed in HCC^23^. In this study, we found that patients with a GT or TT genotype at the rs9642880 SNP had higher incidences of multiple intrahepatic lesions and BCLC C stage. These findings may be explained by the potential overexpression of the oncogene *c-MYC* in the variant genotype. However, further stratified survival analyses revealed that only BCLC stage and not intrahepatic tumour number contributed to the association between the rs9642880 G > T polymorphism and survival of HCC patients. This suggests that BCLC stage has more impact than tumour number on HCC patient survival.

There were several limitations in our study. First, we could not rule out the possibility of selection bias in our study. However, there were no significant genotype distribution differences between the overall population and our HCC cohort^6,24^. Second, although we performed univariate analysis of predictors of survival in 271 HCC patients, and although significant survival prediction factors in the univariate analysis were used to adjust the univariate Cox regression results to eliminate possible interference with the main effects of rs9642880 G > T on HCC prognosis, some potential predictors of survival that were not included may still be a source of bias in the prognosis. Third, in the analyses of associations between rs9642880 G > T and clinical characteristics, there may exist interference among different clinical characteristics, although adjustment was performed.

In conclusion, the study illustrated the potential association between rs9642880 G > T and the survival of HCC patients who received radiotherapy treatment. This association may be attributed to the roles that the variant genotypes play in radioresponse and in the BCLC stage of HCC patients.

## Methods

### Study population

We retrospectively reviewed 271 HCC patients who received radiotherapy at Zhongshan Hospital between January 2009 and October 2014. The diagnosis of HCC was based on guidelines from the American Association for the Study of Liver Diseases^25^. Patients with Child-Pugh class C disease and patients who had a medical history of other cancers were excluded from our study. Clinical characteristics and demographic information were obtained from a review of the medical records. The study was approved by the Zhongshan Hospital Research Ethics Committee, and informed consent was obtained from each patient.

### Therapies

For the 271 patients in the study, conventional external beam radiotherapy(EBRT) was delivered using three-dimensional conformal radiation therapy (3D-CRT) or helical tomotherapy (HT).Briefly, the patients were immobilized by a vacuum-formed mould, which roughly matched the shape of the patients. For HT patients, an anterior pressure plate was added to reduce respiration movement. Gross tumour volume included the whole intrahepatic tumour. Clinical target volume (CTV) denoted the gross tumour volume and an expanded margin of 5–10 mm, and the planning target volume added a margin of 7–10 mm to the CTV to compensate for geometric uncertainties. Treatment plans were established using TomoTherapy planning software, version 4.2 (TomoTherapy, Inc., USA) in HT patients and the XiO treatment planning system in 3D-CRT patients. A daily dose of 2 Gy was administered to every patient at five fractions per week, and the median total dose was 54 Gy (range of 31.2–60 Gy). As much of the normal liver was kept unirradiated as possible. When adverse effects or aggravated performance status occurred, a lower dose was delivered or radiotherapy was stopped.

#### SNP selection

*c-MYC* was identified as a radiosensitive locus. We searched for SNPs in the *c-MYC* enhancer region on 8q24 within the dbSNP database and found three SNPs (rs6983267, rs11986220 and rs9642880) that were previously identified as functional variants of enhancer activity. However, SNPs rs6983267 and rs11986220 are hundreds of kb distal to the *c-MYC* gene. In contrast, the rs9642880 G > T variant is located only 30 kb upstream of *c-MYC*. Therefore, we ultimately selected the rs9642880 G > T SNP for genotyping in our studies.

### Follow-up and response evaluation

The median follow-up time for the cohort was 15.3 months, with a range of 1.5–76.6 months. Patients were advised to receive an initial follow-up examination approximately 1.5 months after the completion of EBRT. The examination included a CT scan, which was performed by doctors who were blinded to the study. Patients were monitored every three months thereafter. Tumour response was evaluated according to the new Response Evaluation Criteria in Solid Tumours (version 1.1)^26^. A complete response (CR) was defined as the complete disappearance of the intrahepatic tumour. A partial response (PR) was defined as a 50% reduction in intrahepatic tumour size. Progressive disease (PD) was defined as an increase of 25% in intrahepatic tumour size. Finally, stable disease (SD) was classified as any response between PR and PD.

### Genotyping

Blood samples were collected from each subject and stored at −80 °C.Genomic DNA was isolated from peripheral blood lymphocytes using a QIAamp DNA Blood Mini Kit (Qiagen, Germany).The rs9642880 G > T genotypes were identified using a polymerase chain reaction-restriction fragment length polymorphism (PCR-RFLP) assay according to the protocol described in our previous study^27^. Briefly, target fragments containing the rs9642880 site were amplified using the following primers: forward, 5′- CCACCACTCTCAGCCTTTTC-3′ and reverse, 5′-TGGGATTACAAGTGTGAACCTG-3′. The 203 bp PCR products were digested by the StyI-HF restriction enzyme (New England BioLabs, USA), and a 2% agarose gel electrophoresis was conducted to identify the rs9642880 genotypes. The genotypes were assessed as follows: the wild-type homozygotes (GG) produced one band at 203 bp; the variant homozygotes (TT) produced two bands at 135 and 68 bp; and the heterozygotes (GT) produced three bands at 203, 135, and 68 bp (Fig. 1a). PCR products from approximately 10% of the samples were randomly selected and verified by direct sequencing using an ABI 3730 DNA analyser (Applied Biosystems, USA), and the results were 100% concordant with the electrophoresis results (Fig. 1b,c,d).

### Quantitative real-time PCR assay

Total RNA was isolated from cells using TRIzol reagent (Invitrogen, USA) according to the manufacturer’s protocol. The reverse transcription-polymerase chain reaction was performed with a ProFlex PCR system (Applied Biosystems, USA). Briefly, 800 ng total RNA was used for cDNA synthesis with a PrimeScript RT reagent kit (TakaraBio, Japan), and real-time PCR was performed with an ABI 7500 detection system (Applied Biosystems, USA) using Hiff^TM^ qPCR SYBR Green Master Mix (Shanghai YEASEN Biotechnology Co.Ltd., China). The expression of c-MYC was normalized to GAPDH expression using the 2^−ΔΔCt^ method, and the primers for human c-MYC were as follows: 5-TCCCTCCACTCGGAAGGAC-3(forward) and 5-CTGGTGCATTTTCGGTTGTTG-3(reverse). The GAPDH primers were as follows: 5-CTGGGCTACACTGAGCACC-3(forward) and 5-AAGTGGTCGTTGAGGGCAATG-3(reverse).

### Protein levels detected by Western blot analysis

Total protein was extracted from cells using Beyotime Cell Lysis Buffer for Western and IP (Beyotime, China) containing protease inhibitors. Proteins were separated by 8% SDS-PAGE and transferred to a methanol-activated PVDF membrane. The membrane was blocked for 1 h in 5% nonfat milk and subsequently probed with an antibody against human c-MYC (1:1500; Cell Signaling Technology, USA) at 4 °C overnight. β-Actin served as a loading control (1:1500; Beyotime, China). Membranes were washed three times for 5 min each with phosphate-buffered saline with 0.05% Tween-20 (PBST) and were incubated with goat anti-rabbit or anti-mouse IgG (1:2500; Beyotime, China) for 1 h, washed three times with PBST and detected with Super ECL Detection Reagent (Shanghai YEASEN Biotechnology Co. Ltd, China). Bands were imaged by the Tanon 5200 Chemiluminescent Imaging System (Tanon, China).

### Statistical analysis

The Cox proportional hazards regression model was used to check for potential predictors of survival among all variables. The multivariate analysis of the rs9642880 G > T polymorphism was adjusted for clinical variables that were significantly associated with overall survival in the univariate Cox proportional hazards regression model. The survival analysis was performed using the Kaplan-Meier method and log-rank test. Associations between the rs9642880 G > T polymorphism and clinical characteristics were calculated using a binary logistic regression model. All statistical analyses were performed using SPSS software, version 20.0 (SPSS Inc., USA).

### Ethical approval

All procedures performed in studies involving human participants were in accordance with the ethical standards of the institutional and/or national research committee and with the 1964 Helsinki declaration and its later amendments or comparable ethical standards. This article does not contain any studies with animals performed by any of the authors.

## Electronic supplementary material

Supplementary information
