# Supplementary material for: A sequence polymorphism on 8q24 is associated with survival in hepatocellular carcinoma patients who received radiation therapy
Source: Sci Rep. 2018 Feb 2;8:2264. doi: 10.1038/s41598-018-20700-x (PMC5797243; doi:10.1038/s41598-018-20700-x)
Supplement: Supplementary file 1 — Supplementary information [file 41598_2018_20700_MOESM1_ESM.pdf]

A sequence polymorphism on 8q24 is associated with survival in hepatocellular carcinoma patients who received radiation therapy

Xiao-Mei Zhao[1], Zuo-Lin Xiang[1], Yi-Xing Chen[1], Ping Yang[1], Yong Hu[1] and Zhao-Chong Zeng[1*]

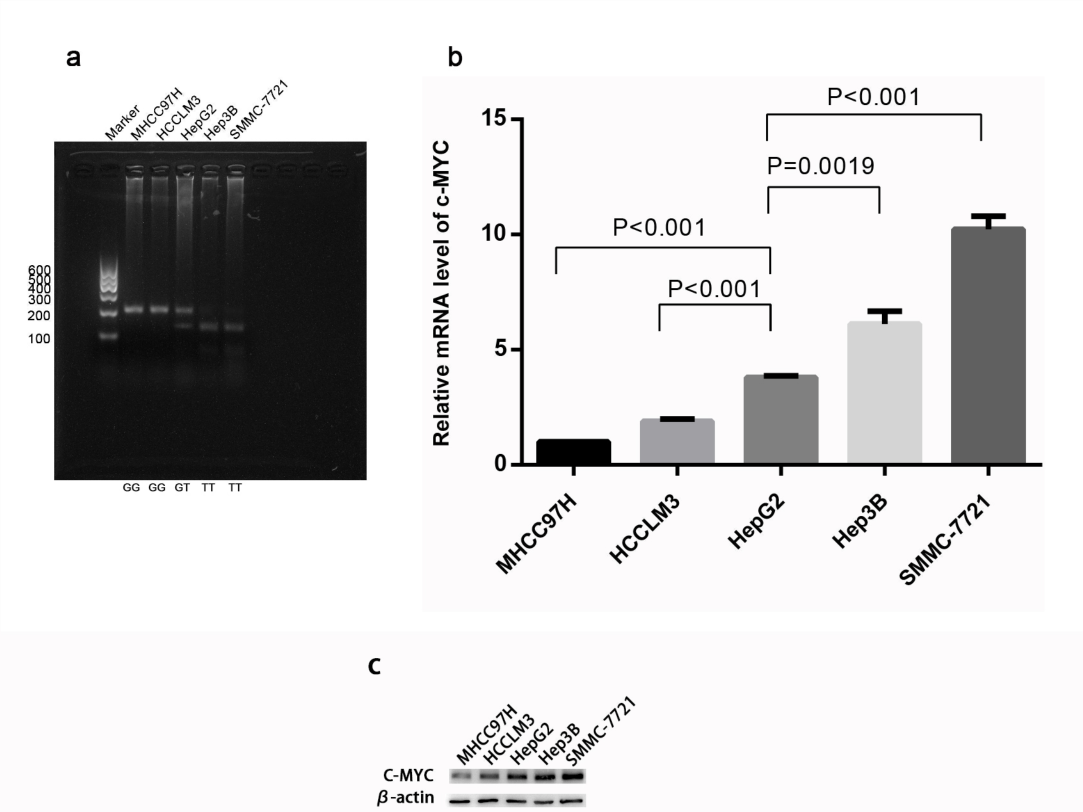

Supplementary fig.1. Rs9642880 G>T polymorphism genotypes of HCC cell lines (MHCC97H, HCCLM3, HepG2, Hep3B, SMMC-7721) were identified using the PCR-RFLP assay. The c-MYC mRNA levels of the HCC cell lines were detected using quantitative real-time PCR. a: HCC cell lines,MHCC97H, HCCLM3 had the GG genotype, HepG2 had the GT genotype, and Hep3B and SMMC-7721 had the TT genotype. The figure of the full-length agarose gel and figure with white background and black product bands is included in the supplementary fig 2b and 3b; b: c-MYC mRNA levels of HCC cell lines. Data are presented as the mean ± SEM and are representative of three independent experiments.; c: c-MYC protein levels of HCC cell lines.

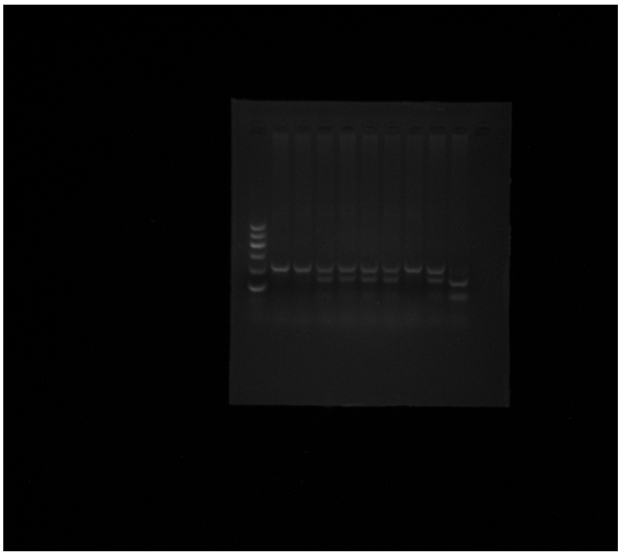

Supplementary fig.2a. The full-length agarose gel of fig 1a.

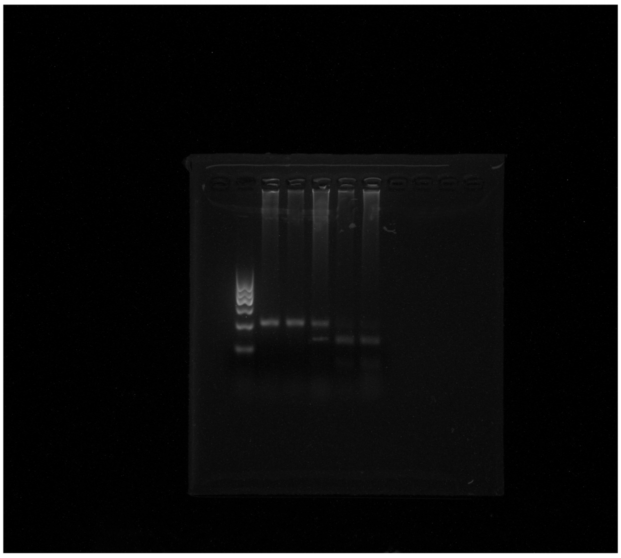

Supplementary fig.2b. The full-length agarose gel of supplementary fig 1a.

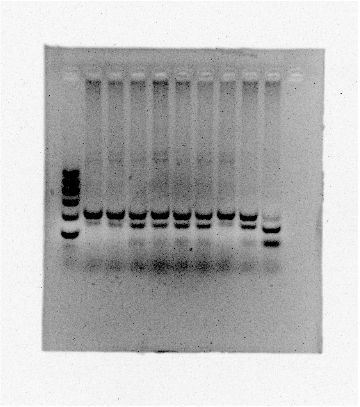

Supplementary fig.3a. The figure with white background and black product bands of fig.1a.

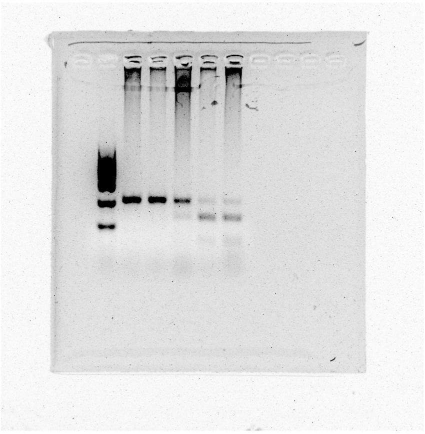

Supplementary fig.3b. The figure with white background and black product bands of supplementary fig.1a.
